# Pathologies of imagination and affectivity: the genesis of the unconscious in Marc Richir

**DOI:** 10.3389/fpsyt.2023.1197937

**Published:** 2023-06-30

**Authors:** Mauro Senatore

**Affiliations:** Department of Philosophy, Universidad Adolfo Ibáñez, Santiago, Chile

**Keywords:** imagination, affectivity, empathy, Edmund Husserl, Marc Richir

## Abstract

In their recent work in phenomenological psychopathology, Andreas Rosén Rasmussen and Joseph Parnas argue that there is an expressive relationship between the anomalies of imagination reported in schizophrenic spectrum disorders (SSDs) and an underlying generative self- or ipseity disorder. The authors build their argument on an updated review of the phenomenological model of consciousness, by which each experience articulates itself in ipseity according to its modality. Therefore, they explain imagination as the figuration of an absent object mediated by the imaginary and accompanied by a sense of irreality. Finally, by drawing on patients' descriptions, Rasmussen and Parnas show that SSD imagination disorders testify to the breakdown of this model of consciousness. In this article, I aim to complexify the scenario summarized above by focusing on the contribution made by the phenomenologist Marc Richir in his late masterwork Phantasia*, imagination et affectivité* (2004). To this end, I examine the genetic analyses of the pathologies of the imaginary that Richir develops through a non-standard interpretation of Husserl's phenomenology of imagination (in particular, Hua XXIII, text n.16, 1912). In my examination, I aim to unfold an alternative model of consciousness that (a) is based on the gap between the architectonic registers of *phantasia* and imagination (and the corresponding stages of sense-making and the institution of sense), (b) takes account of the role of affectivity in those registers, (c) places the pathologies of the imaginary in the quasi-empathy that characterizes the missed encounter with the other, and (d) links the institution of these pathologies with the psychoanalytic account of the fixation of the phantasm.

## 1. Introduction

“Passing into the image (*hinein ins Bild*), as Husserl puts it, is first of all passing into the *Bildobjekt* or ‘perceptive appearance,' it is for consciousness losing its own intentionality therein, no longer seeing imagined objects, but only ‘thinking' of them emptily [à* vide*]” (PIA 34)^1^

In their recent work in phenomenological psychopathology, Andreas Rosén Rasmussen and Joseph Parnas draw attention to the anomalies of imagination that are characteristic of mental disorders such as schizophrenic spectrum disorders (SSDs). In particular, they argue that there is an expressive relationship between these anomalies and an underlying generative disorder of the self, the so-called self- or ipseity disorder (1). To develop their argument, they put forward a model of consciousness in which the traditional phenomenology of imagination [from Husserl to Sartre; (2)] is reinterpreted in light of the theory of the minimal self or ipseity (3, 4), here reviewed as the first-person perspective in which all lived experiences articulate according to their intentional modality (5, 6). In the case of SSDs, by building on the phenomenological material represented by the descriptions of patients, Rasmussen and Parnas show that the anomalies of imagination testify to the breakdown of this model of consciousness.

The authors start from the following definition of imagination, “as an experience of ‘inner' mental visual images with a prereflective awareness of their unreality” [(1); 318], which they develop by resorting to the phenomenological tradition. On the one hand, they recall that, for Husserl, “phantasy” (*Phantasie*; a term that they employ as a synonym of imagination) represents an absent object (*Bildsubjet*) through a certain appearance of another kind of object, namely an image (*Bildobjekt*). This does not mean that we experience two objects; more accurately, we experience the absent object in/through the image. As the authors remark, “the image cannot be understood as a picture in my mind,” or, in other terms, it cannot be apprehended as such, “like a perceptual object”; rather, it consists in “an intentional medium or relation of consciousness to an absent intentional object” [320; (7)]. A cardinal point here, according to the authors, is that “whereas the image is present to consciousness, the represented content of phantasy is immediately, prereflectively given as non-present,” in contrast to what occurs with a perceived object [(1), 319]. By this prereflective awareness, we should understand an immediate sense of the intentional modality that is distinctive of imaginary experience. On the other hand, Rasmussen and Parnas explain this cardinal point by appealing to Sartre's theory of the imaginary. They subscribe to the idea that “the image is experienced as irreality, i.e., one is prereflectively aware, *in the very act of imagination*, that the experience has another kind of ‘reality status' than a *perceived, present* object” (319; 8). Finally, they situate imagination in a model of consciousness in which all modes of intentionality are prereflectively self-aware of their specific modality, namely, are “given in the first-person perspective as *my experience of a certain kind*” [(1); 319]. To summarize, when I imagine an absent object, I do not require a reflective act to know that I am not perceiving a present object.

Within this framework, Rasmussen and Parnas classify the anomalies of imagination reported by their patients into the following three categories. First, they speak of a “perceptualization” and “spatialization” of the image. As they explain, the imagery has become explorable by itself and liable to detailed descriptions. Moreover, it flows with a certain independence from the willingness of the patient who, thus, is turned into a spectator (320). Second, the increasing perceptualization of the imagery entails an intensification of the affective response to the picture with respect to the poverty and superficiality that characterize the affective response to images as compared to perceptual objects (321). Third, we assist in the erosion of irreality, that is, of the sense of irreality that is supposed to determine the intentional modality peculiar to imagination. In the cases examined by the authors, the experience of imagery is given as present and is not distinctively detached from the perceptual world (321). Now, these anomalies reveal, through the disturbance of a particular kind of intentional modality (imagination), the more general breakdown of the model consciousness in which this modality is inscribed. As the authors put it, they are “a particular kind of disturbance of [...] the sense of self or ipseity,” which here is understood as “the first-person perspective in which all experience articulates itself” as an experience of this or that kind. In this case, the disorder of ipseity compromises (i) this dynamic interpenetration of the imaginary object with the first-person perspective in which it is given (322) and, ultimately, (ii) the prereflective self-awareness underpinning each intentional modality of my consciousness.

In this article, I aim to account for the contribution to the trend in phenomenological psychopathology described above that, in my view, is made by the poorly known genetic analyses of mental pathologies unfolded by the phenomenologist Marc Richir in his late masterwork Phantasia*, imagination et affectivité* ((8); hereafter indicated as PIA).^2^ The Richir scholarship has already drawn attention to this contribution. In a recent article, (11) bring Rasmussen and Parnas's analyses into conversation with what they designate as an imaginary turn in phenomenological tradition and illustrate by referring to the phenomenology of embodied *phantasia* that Richir had developed since *Phénoménologie en esquisses* [(12), hereafter indicated as PE]. If this turn shares with those analyses its focus on the anomalies of imagination in mental pathologies, however, it calls for an alternative explanation of these pathologies on account of an alternative conception of imagination (not as an intentional modality of consciousness among others). As Fazakas and Gozé explain, Richir builds on an original interpretation of Husserl's 1904–05 analyses of imagination to develop a conception of *phantasia* (i) as the most archaic and preintentional structure of consciousness, underpinning the genesis of imagination and perception, and (ii) as embodied (*Phantasieleib*), that is, as anchored in its body schema and corresponding kinesthetic habits (PE 72–143). Within this alternative framework, they argue that the anomalies of imagination are not expressive of more fundamental ipseity disorders but of a certain deformation of the embodied *phantasia* (namely, the *Phantomleib*).

In what follows, I aim to bring these analyses further precisely by focusing on Richir's complex account of this deformation, which, as I will explain, corresponds to the institution of a-subjective imaginative intentionalities in the *Phantomleib*. As we see later, although this institution shifts from the deformation of *phantasia* into imagination, which Richir identifies as the former's architectonic transposition into the latter, certainly its implicit possibility can be found in that transposition (and, more accurately, in the non-positionality of the *Bildobjekt*, or the imaginary).^3^ In other words, in Richir's view, here, we have to do with pathologies whose germs are already at work in the institution of imagination.^4^ Thus, I would like to show that Richir's contribution to the phenomenological psychopathology of imagination amounts to providing us with an original genetic analysis of the psychoanalytic unconscious that develops from his non-standard interpretation of the Husserlian phenomenology of imagination (see the Introduction to PIA) and undergirds his phenomenological rewriting of Freud's exploration of mental pathologies (neuroses and perversions; see PIA Section 3).^5^ It is from this perspective that, here, I cast light on the first chapter of the Introduction to PIA, where Richir traces the beginning of the *Spaltung* (which, for reasons that become evident below, corresponds to the genesis of the unconscious) in the aporetic version of his phenomenology of imagination that Husserl offers in Hua XXIII, text n.16 (18).^6^ In this chapter, Richir starts from a passage from Husserl's text, which examines what happens when a situation of sadness hovers in our imagination (*Phantasie*), in order to explain that there is a single structure of the *Spaltung* (with two different entrance points corresponding to the two cases of affectivity at stake there) which takes place in the structure of imagination. Moreover, for Richir, the institution of the *Spaltung* causes the fixation of the psychoanalytic phantasm, which he understands as the complex of significativities meant by the aforementioned a-subjective imaginative intentionalities.^7^

To test my reading hypothesis concerning Richir's genetic analyses of the unconscious, in the next sections, I will focus on the following three steps of his interpretation of Husserl's text:

(i) The analysis of the first case of affectivity examined by Husserl (the affectivity that I imagine by projecting myself in the situation of sadness). In this case, we will see that Richir finds the beginning of the *Spaltung* when the image fascinates me and thus when the non-positionality of the *Bildobjekt* is cleared of the quasi-positionality of the *Bildsujet* (namely, it becomes absolute non-positionality) and the life of the imaginary I passed into the images exceeds that of the imagining I. Here, for Richir, there is only one step left for the transition of this kind of *rêverie*, or daydreaming, into that which Freud designates as “unconscious thoughts,” that is, the coupling with an affective excess that prevents those thoughts from being accomplished in/by consciousness as a fiction;

(ii) The analysis of case 3 (the affectivity felt when I stand before the imagined situation). In this case, I will show that the *Spaltung* begins when an imagined scene is felt with the character of a mood (*Stimmung*) that cannot be localized in it and, rather, is retained in the *Bildobjekt*. This atmospherized mood has the atmospherization of a part of the imagining I into non-localized imaginative intentionalities as its correlate. In this case, Richir sees the unconscious at work in the part of the *Bildobjekt* invested by the mood and in the corresponding a-subjective intentionalities;

(iii) The reinterpretation of these cases as pathologies of empathy (*Einfühlung*). We will see that, for Richir, the empathy carried out through imagination, a quasi-empathy, in which I feel the internal life of the other through the image that I have of it, is pathological as it overlaps with the pathologies of affectivity and imagination at stake in the two cases evoked above. Through this reinterpretation of imaginative empathy, Richir offers a recapitulation of the *Spaltung* as a global structure that encompasses the aforementioned cases and develops as a circulation of affectivity through them.

In the conclusion, I will draw out of my exploration the relevance of Richir's analyses to the phenomenological psychopathology of imagination discussed above. I will explain this relevance as follows: (1) a remarkable complexification in the account of these aspects of the life of consciousness: (a) the architectonic relations between *phantasia*, imagination, and pathological imaginative intentionalities; (b) the difference between empathy and the quasi-empathy pursued through imagination; and (c) the role of affectivity; and (2) the project of interweaving phenomenological psychopathology and psychoanalysis, which, although I cannot discuss here in great detail, can be found already at stake in the articulation between the institution of the phenomenological *Spaltung* and the fixation of the psychoanalytic phantasm.

## 2. The genetic analysis of the unconscious

Richir's overall interpretation of Husserl's text unfolds as a selective close reading through which Richir searches for the beginning of the *Spaltung* sketched and yet left undeveloped by Husserl. I will not follow this reading punctually, but I will engage in a comparative discussion of Husserl's argumentative steps and Richir's interpretative remarks, which aims to retain the trajectory of Richir's reading and highlight the latter's demarcations from its source. Richir's point of departure is the passage from text n.16 in which Husserl identifies the three different cases that can occur when I imagine a sad situation.^8^ For Richir, this text provides us with the description of the aforementioned global structure of the *Spaltung*. Husserl writes:

In the same way, if a sad situation hovers before me in phantasy, then either the grief belongs to the phantasy, namely, when I project myself into the nexus of the phantasy and do so as one who is grieving (I stand and grieve, for example, at the bier of someone presented in phantasy as deceased); or if I do not phantasy myself and my grief into the sad situation but instead phantasy someone else who is grieving, then it is his grief that is phantasied; or, finally, I do not phantasy any grief whatsoever, but actually “sense” grief on the basis of the presentation (PIA 9; PICM 554–5).

The three cases broken down here can be summarized into the following two: either the grief belongs to imagination, when I project myself (or another) into the imagined situation and thus as grieving; or I feel the grief by standing before the imagined situation.^9^ Richir's interpretation of this passage starts by displaying its non-standard phenomenological framework, namely the architectonic account of consciousness that Richir had developed in PE and for which, alternatively to standard Husserlian scholarship, he divides Husserl's *Phantasie* into *phantasia*, as the most basic moment in the life of consciousness, and *imagination* (understood as without the support of a physical image and thus as different from image consciousness), which results from the architectonic transposition of *phantasia* and is characterized by objective intentionality (PE 61–134).^10^ For Richir, here Husserl employs *Phantasie* in the guise of imagination (as Richir puts it, “imagination has already been instituted”) to the extent that “in each case, we have an intentionality aiming at an object” (PIA 9). Furthermore, Richir highlights in Husserl's text the division of affectivity into two kinds that correspond to the two gates of the structure of *Spaltung* to be examined: (i) an imagined grief, which presupposes that I pass entirely into the imagined situation (as myself or an imagined other) in order to feel it; (ii) a felt grief, which implies that I keep at a distance from the imagined situation and feel the grief effectively as if the latter were real (21, 25). In what follows, Richir builds on Husserl's analyses of the phenomenological status of the imagined situation and the imagining I in relation to each kind of affectivity, in order to develop his genetic exploration of the *Spaltung* and the unconscious.

### 2.1. The case of figured affectivity

Husserl starts from the case of imagined affectivity. He takes account of the implications of the self-projection in the imagined situation (*Bildobjekt*): the duality between the actual (imagining) I and the image (imagined) I (*Bild-Ich*) and between their corresponding lived experiences. In particular, he is interested in the question of the phenomenological status of the *Bildobjekt* and the corresponding appearance (of which the image I is a part), namely, of their non-positionality. How we can conceive of the latter in relation to the internal time consciousness in which all lived experiences are posited, Husserl wonders. He pushes his exploration so far as to question himself concerning the possibility that the non-positionality of the image object be absolute, or, that an alteration of consciousness be carried out such that I apprehend the imaginative apprehension by itself, that is, as a kind of merely perceptive and non-figuring apprehension. As I will show, Richir finds in the non-positionality of the *Bildobjekt*, for which it escapes time consciousness and, eventually, fascinates me, the implicit possibility of the *Spaltung* and the basis for his first genetic hypothesis about the origin of the psychoanalytic unconscious.

First, Husserl explains that my self-projection into the image consists in assimilating myself into the image and putting my actual I out of service, which means becoming non-positional like the *Bildobjekt*. Interestingly for the development of this analysis, Husserl describes this case in terms of a special kind of empathy. He remarks that here the participation in the imagined situation of affectivity, namely, empathy, occurs *in* and not *in front of* the image (and, thus, in relation to the imagined object; PIA 10; PICM 556). What are the consequences of this self-projection, for Husserl? As I anticipated, here, we have a duality of I's, the I of the phantasy world (given that this world presupposes a center of orientation) and the actual I (that is the subject of the act of imagination), which raises the question about how the lived experiences belonging to each I, respectively, are related (PIA 11–12; PICM 556). As Husserl points out, the I in the image is at once non-positional and perceptive (PIA 13; PICM 557), in the sense of the perception (*Perzeption*), alternative to the actual one (*Wahrnemung*), that he had recognized earlier on as distinctive of the I immersed in the image (PICM 555–556). Evidently, it is here that Richir finds the first possibility of the *Spaltung* implicit in Husserl's treatment of the image. In his remarks on Husserl's analysis, Richir focuses on the character of non-positionality that the I passed into the image takes from the latter. For Richir, it is precisely in that non-positionality and thus in the fact that the latter cannot be completely covered by the quasi-position of the object imagined by the actual I that the *Spaltung* takes place. Richir writes:

If we understand that the I in the image does not posit, should we put this absence of position into relation with the quasi-position of the imagined object (*Bildsujet*) in the imagination? Or, rather, does the quasi-position depend on the actual I that (quasi) posits the imagined object through the image and, in this case, is there not a *Spaltung* of the I, between the I that does not posit and the actual I that posits? What happens if the actual I is lost (*selbstverloren*) in the imagination? Is there still something to “contemplate” in that which would be a “life” that has entirely passed into the image? (PIA 11)

Furthermore, for Richir, as it develops, Husserl's analysis confirms this interpretation in an explicit way. By positing the duality of the lived experiences belonging to the Is under scrutiny, Husserl seems to acknowledge that a part of the lived experiences of the actual I can pass into those of the I in the image, and thus, the latter leads its own lived experience beyond the life of the actual I (PIA 12–13).^11^

The next step of Husserl's analysis consists in measuring the question of the non-positionality of the image and the I absorbed in it against the axiom of time consciousness, in which all lived experiences are posited or can be accomplished through a reflexive act. In the case of image consciousness, Husserl explains, the latter, which, as we know, is at once perceptive and non-positional, is still posited in time consciousness. Therefore, the same must hold for the I in the image. It is worth highlighting that, for Husserl, the experience posited in time consciousness here is the perceptive consciousness of the non-positional I as belonging to the image (as he puts it, the experience in which “one is perceptually conscious of the Ego in a non-positing way as a member of the image world”). From this, Husserl concludes that “by being posited in internal consciousness, the self-perceiving is obviously not *eo ipso* a positing of itself as reality, as one might think” (PIA 14; PICM 558). Richir's remarks linger on this point since, on his reading, it allows for the possibility that the imaginary I can be posited internally as such and thus leads its own life, excessive with regard to consciousness. In Richir's words, “the I passed into the image cannot perceive itself but... as *an imaginary self* that is not effectively real in the internal consciousness and, therefore, somehow escapes it” (PIA 14). I recall that here Richir replaces time consciousness, which, constitutes the basic structure of the standard Husserlian-type consciousness, with the immediate transcendental apperception (hereafter referred to as ITA), which, in his non-standard genetic phenomenology, accompanies the more archaic layer of *phantasia* and refers to the ipseity of the sense making itself in this layer (PIA 434–35). Therefore, for Richir, in this passage, Husserl describes the situation of “a possible sliding of ITA” (PIA 14–15). Here, Richir seems to echo the Sartrean lexicon by describing the dissociation between the non-positionality of the imaginary I and the quasi-position of the act of imagination, that is, the becoming radical of that non-positionality, as a case of *fascination* by images.^12^ Therefore, Richir observes that, for the sliding of ITA to occur, we just need that “the image exercises a power of fascination that makes us forget the entire act of the imagination in which the lived experience of imagination is posited” (PIA 15).

Husserl pushes his examination further by unfolding the comparative reading of felt and imagined affectivity that he had sketched above in relation to empathy. In doing so, he raises a key question concerning the relation between affectivity and imagination: what does to imagine or to figure an affect mean? As we see in a moment, Richir reads these remarks from the perspective of phenomenological psychopathology as he implicitly interprets the cases examined by Husserl in terms of anomalies. Furthermore, Richir casts light on the anomalous kind of empathy that is at work therein and, consequently, lays the ground for his later reformulation of the pathologies of empathy. Thus, Husserl returns to the initial situation of affectivity hovering in imagination, in which we recognize that two kinds of affectivity can be at play. As we learned from the analysis developed so far, in both cases, affectivity is modified, non-positional, and yet internally posited. Here, Husserl demarcates the two kinds of affectivity from one another on the basis of their being figured or not. In case 1, as Husserl recalls here, affectivity is figured, in the sense that I imagine myself as feeling delight while I am absorbed in the image. In case 3, affectivity is non-figured as I feel it effectively by facing the image (PIA 15–16; PICM 560). As we see later, it is more properly a mood (*Stimmung*), that is, a non-localized and atmospherized kind of affectivity [(21), 617–6199]. Husserl ends this analysis with the following tentative conclusion, which, in his view, remains to be explained:

In the one case, however, the non-positing delight itself synthesizes with the image consciousness and belongs to its composition; in the other case, it does not. In the first case, a delight is exhibited in the non-positing delight, just as a person who is ill is exhibited in the non-positing appearance of a person who is ill. In the other case, I have a non-positing delight, but nothing exhibits itself in it (PIA 16; PICM 560).

As I observed, Richir finds in this passage the indices of the transcendental pathologies of imagination *and* affectivity. First, Richir explains the difference between felt and figured affectivity by emphasizing the relation between affectivity and the imaginary. In case 1, the affect belongs to the image, he observes, and thus, I can feel it only by passing entirely into the image. In case 3, the affect belongs to the lived experience of the actual I and cannot be found in the image as being figured in it (PIA 16). Although Richir does not develop this interpretation in an explicit way, here we can see, I suggest the structure of the *Spaltung* at work in each case: either affectivity belongs to the life of the imaginary I or the actual I facing the image feels it without knowing why, that is, without grasping the link between affectivity and the imagined object. Later, Richir unfolds the context of the intersubjective relation in which Husserl's analyses implicitly take place and, more precisely, of the intersubjective relation mediated by imagination, thus without the other, given that imagination, as Husserl himself argues elsewhere, bears an element of self-projection or narcissism that prevents me from feeling the lived experiences of the other [(28), texts n. 10 and n.13; PE 267–91]. In doing so, Richir links the pathologies of imagination and affectivity that we had highlighted above with the pathological character of imaginary empathy, which he develops more profoundly later. Therefore, he evokes the two following kinds of development corresponding to the cases of affectivity described by Husserl:

Figuring or representing affectivity, so it seems, can only signify “imagining” *in* the image, through a sort of strange *Einfühlung* without the other, a *quasi-Einfühlung* (in psychological terms: projection) that, if carried out, paradoxically makes the actual I relatively insensible to itself (the actual I does not live properly the imaginary lived experiences), and, if not carried out, puts the actual I into an affectivity that it feels but does not figure to itself (by *Darstellung*) in the image (PIA 16–17).

Here, Richir explicitly finds something of the *Spaltung* at stake (PIA 17). According to his psychopathologically driven interpretation of Husserl's text, the pathologies of empathy, in which imagination and affectivity are interwoven together, would be of two kinds: when the supposed or imagined affectivity of the other is felt only by the imaginary I and thus in dissociation from the actual I; or when I feel it effectively in my lived experience but cannot find it in the image and thus cannot explain it.

Husserl addresses this question about the difference between figured and non-figured affectivity by further exploring the concept of image consciousness, which he had explained earlier on in this text as a perceptive and non-positional experience. As we see in a moment, this further exploration does not offer an answer to that question but brings more questions to the stage. We can distinguish two moments in Husserl's reconsideration of image consciousness. First, he remarks that the image appearances that inhabit the world of imagination (image things, plants, and animals) are not merely perceptive but also imaginative, in that, by definition, they figure another appearance, which is absent (PIA 17; PICM 560–561). This remark does not sound so innocent if we consider that it draws attention to a kind of a-subjective imaginative intentionality that is cut from the imagining I. As Richir observes, interestingly Husserl seems to admit that the imaginative appearances have their own intentionality and thus that the actual I bearing the intentionality of imagination is put out of service. “Should we speak of *imaginative intentionalities* without an actual subject?”, Richir asks (PIA 17). Of course, we are not in the case of the *Spaltung* since here we have no absolute non-positionality at play yet. However, Richir's remark is worth recalling since a-subjective imaginative intentionalities play an important role when, as we see in a moment, the imaginative appearances are apprehended as such, and thus, imagination is no longer instituted on the basis of *phantasia* and the *Phantasieleib* but on the lived body related to this kind of apprehension that Richir designates as *Phantomleib*.^13^ Second, Husserl puts forward a paradoxical hypothesis that vouches for the absolute non-positionality of the imaginative appearance and the beginning of the *Spaltung* between the imaginary I and the actual I. He wonders if “we can take... the apprehension as nonexhibiting apprehension,” and thus if “we can execute a change of consciousness that therefore *carries out* the apprehension as perceptual [*perzeptive*] apprehension” (PIA 18; PICM 561). This hypothesis does not only allow Richir to clarify the phenomenological status, that is, the non-positional character, of the *Bildobjekt*, but also indicates the point where this character can turn into absolute or radical and open the field of mental pathologies. It is at this point that Richir formulates his first genetic hypothesis about the articulation of phenomenology and psychoanalysis and the origin of the unconscious. Richir argues that we can find in the Husserlian phenomenological account of the *Bildobjekt*, that is, in the acknowledged possibility of its absolute or radical non-positionality, also the possibility of the *Spaltung* that we have been tracking so far. According to Richir's reading of Husserl's text, the absolute non-positionality of the image object consists in the detachment of the life of the imaginary I from that of the actual I, or, in other words, in the case of imagined affectivity and empathy (16). Thus, we are at just one step from the psychoanalytic unconscious, which, as we see later, requires the interweaving of this situation of *Spaltung* with a traumatic affectivity (an excess or deficit of affectivity in the encounter with the other). Richir writes:

The non-positionality and evanescence of the lived experience are the beginning of one of the figures of the *Spaltung*, that of neuroses in general, which Freud conceived of as “unconscious thoughts.” A beginning because the passage from non-positionality to the unconscious would still be required [...] This passage would have as a remarkable correlate the fact that the intentionality of imagination would be empty of imagined objects since then, and thus it would have no longer imagined objects susceptible of being intuited, given that the *Bildobjekt* or the “perceptive appearance” would be completely autonomized. We would have to do with pure or empty imaginative intentionalities which only mean significations or *significativities* (*Bedeutsamkeiten*) that are *coded* from somewhere else, in a state of non-positionality, of not being accomplished by consciousness, that is, of *unconsciousness*, since nothing is posited by consciousness. To speak the language of psychoanalysis, this would be the case of “phantasm” (PIA 19).

Prior to turning to Husserl's analysis of case 3 (affectivity and empathy felt without an explanation), which provides Richir with the basis for his second genetic hypothesis about mental pathologies, I would like to draw attention to the final part of this text. On the one hand, Richir highlights the kind of imaginative intentionalities that are at work in the *Spaltung* and the unconscious, which do not merely overlap with those suggested by Husserl in the aforementioned passage. On the other hand, Richir describes the complex of significativities that are aimed at by those intentionalities, namely what he calls “phantasm” in the wake of psychoanalysis, as “coded,” that is, as implying the intervention of the Freudian “primary process.” As Richir explains later, in his phenomenological rereading of the psychoanalytic account of mental pathologies, the primary process, which contributes to the fixation of the phantasm, consists in an a-subjective and parasitic process of distortion of sense (through the condensation or displacing of *phantasia* apparitions), which draws on the habits and sedimentations that are culturally inherited by the subject.^14^

### 2.2. The case of the non-figurative mood

Husserl engages in a further examination of case 3 in the last fragment of text n.16, entitled “Exhibiting of feelings in the image as moods (not as personal feelings).” The fragment deals with a special kind of affectivity, a mood (*Stimmung*), that merges with the affectivity at stake in case 3, which is effectively lived and yet cannot be found in the imagined scene. This mood (a) does not belong to a character figured in the scene and, therefore, (b) its correlate is not the I involved in the act of imagination (neither the imagining/actual I nor the imagined/imaginary I within the image object) but enters the scene precisely as such, atmospherized and unlocalized like the corresponding mood. Despite this last feature, Husserl advances the hypothesis that it is posited by imagination and thus is the result of a quasi-position. Richir shifts from his source precisely on this point. By placing the mood within the architectonic framework of his non-standard genetic phenomenology, he argues for the possibility that it belongs to the *Bildobjekt*. In this case, it may be awakened for the actual I but the latter would not know why and how. Evidently, this interpretation also raises the question concerning the archaic implication of affectivity with consciousness and of the metamorphoses of affectivity across architectonic registers in Richir's non-standard genetic phenomenology. For Richir, the mood is meant by a-subjective imaginative intentionalities, that is, by intentionalities that are dissociated from the imagining I and presuppose the atmospherization of a part of it, namely the *Phantomleib*. We can see that this interpretation leads us back to Richir's earlier analysis of case 3: I find myself in a situation in which the affectivity awakened by an imagined scene and, consequently, a certain fascination that this scene exercises over me escape consciousness and almost betray another life beyond imagination. Richir builds on this interpretation to put forward his second genetic hypothesis about the unconscious.

As I anticipated, here Husserl centers on the figuration of a special affectivity, which is not that of figured characters but constitutes the non-figurative element of the figured scene, as it cannot be found in the latter, and has as its correlate an I that is called into play precisely as such. Husserl writes:

A landscape awakens a mood. A picture of a landscape presents a landscape in a mood: In looking at the image, I do not need actually to get into the mood. Such exhibited moods, feelings, and so on, do not presuppose a co-exhibiting of the spectator, although the spectator goes into action in his own way. More precisely, I, with *this* mood, certainly do not belong in the picture. Should I say: I, not as an empirical human being, but “purely as the correlate of the mood”? (PIA 23; PICM 565–66).^15^

As Husserl points out, this mood is effectively felt and yet belongs to the lived experiences of neither the imagining I looking at the imagined scene nor an imaginary I passed into it. An I springs as the correlate of this mood, or, as indissociable from the awakening of the latter with the imagined scene. What is the relation between this mood and the scene? How is the mood lived and intended? Husserl addresses these questions by advancing a genetic hypothesis that, from Richir's perspective, does not take account of all the possibilities that are implicit in the described situation. Husserl suggests that the mood under scrutiny is the object of a position by imagination and thus that the intentionality meaning it is still that of an imagining I:

The mood is a *quasi*-positing act that bestows on the landscape the ontic mood. The landscape is a landscape exhibited *with* this ontic characteristic. The mood exhibits itself in my *quasi*-mood. In my *quasi*-being-in-a-mood, I am conscious of the mood of the landscape (as of a *quasi*-mood); and my *quasi*-being-in-a-mood exhibits to me the mood of the landscape (PIA 24; PICM 566).

Furthermore, Husserl makes it explicit that the affectivity examined here does not belong to the characters figured in the scene. In this case, we would have the kind of empathy that is at play among actors, spectators, and the figured character in a theatrical representation, which Husserl explores elsewhere (Hua XXIII, text n.18; PIA 498–507). Alternatively, here Husserl focuses on those moods that are “characteristics of exhibited things and are themselves exhibited characteristics,” and, on the other hand, “do not belong to exhibited persons as their experiences, thoughts, and so on” (PIA 25; PICM 566). As we may suppose, Husserl is speaking about a *figured* mood in the sense that he had hypothesized earlier on, that is, as the object of the quasi-position of an imagining I.

Richir takes Husserl's analysis as his point of departure for reinterpreting case 3 as the other gate to the structure of the *Spaltung* and thus for casting light on another pathological articulation of imagination and affectivity. To this end, he argues that the mood in question admits the possibility of another genetic explanation. Therefore, he brings the possibility of the mood back to the *Spaltung* between *Phantasieleib* and *Phantomleib* that would be implicit in Husserl's analyses. First, Richir explains that I am intentionally implicated with the mood as the latter's correlate and then I must be non-localized and volatile like the mood itself, given that it cannot be found in the figured scene (PIA 23). This I is anchored in the *Leib* or the *Phantasieleib* (like the imagining or imaginary I), precisely because the mood does not require a center of orientation (PIA 23). Second, Richir unpacks his alternative explanation of the origin of the mood and its correlate, which pushes further the *Spaltung* between *Phantasieleib* and *Phantomleib* at stake here. If we assume that the mood is the non-figurative and floating element of the imagined scene and that the *Phantomleib* is the part of the *Phantasieleib* that has been atmospherized, then the mood may depend on the *Bildobjekt* first and, later, its character may be transferred to the imagined scene. In this case, the mood would operate as a kind of colored filter, in an unexplained way. As we can understand, Richir's alternative genetic account also consists of a genetic hypothesis about the unconscious. He writes:

It goes without saying that the *Leib* as well as the *Phantasieleib* can really *feel* some *Stimmung* not, again, as exclusively related to them as a (subjective) source, but in relation to the quasi-posited *Bildsujet* that receives the character of the *Stimmung* and, at the same time, by an architectonic transposition, the latter can be caught in the [*prise au*] *Bildobjekt* [...], “vaporize” or “atmospherize” in the *Phantomleib*, that is, be caught in an “affect” that is not lived (not accomplished in the lived experience), like a diffused coloration (a colored filter that is not conscious as such), but not a real (*real*) one, of the landscape (of the *Bildsujet*) (PIA 24).

In this case, the intentionalities meaning the mood are dissociated from the intentionality of the imagining I and from intuitive objects; thus, the mood is not a quasi-position, which, as we know, is the only possibility admitted by Husserl (PIA 24). The *Spaltung* between *Phantasieleib* and *Phantomleib* becomes evident in that my *Leib* or *Phantasieleib* can feel the mood in relation to the imagined scene that is filtered through it but are not there as their correlate. As Richir puts it, affectivity is thus the privileged site of the *Spaltung* (PIA 25). If in case 1, the imagined scene fascinates the I that passes entirely into the image and feels the figured affectivity; here, in case 3, I am fascinated by the imagined scene without an explanation, or unconsciously, as it bears the character of a mood that is not figured in it. In the latter case, a certain affectivity must have transferred into the *Bildobjekt* and must have presupposed the atmospherization of the I as its correlate. As Richir explains later, the institution of imagination that is at stake here consists of an instantaneous arrest of the intersubjective sense-making. This arrest hinges on an excess of affectivity, not necessarily a traumatic one, that interrupts the play of *phantasia*, in which affectivity is stabilized and harmonized, and thus is transposed into the *Bildobjekt* [PIA 443–45; (25), 75–91].

Third, Richir builds on the last part of the examined fragment to establish the architectonic role of the *Phantomleib* more precisely. He starts by transcribing into his lexicon Husserl's demarcation of the awakening of the mood from the empathy with that which the characters figured in the scene are supposed to feel and think (PIA 25). Drawing on this distinction, Richir also clarifies the difference between imagination and *Phantomleib*, which, as we see later, is full of consequences for his interpretation of the pathologies of empathy. What may be at stake here is not a healthy empathy, not even the one carried out by imagination on the basis of *phantasia*, but a pathological one. As Richir explains, in this case, the *Phantasieleib* may be responding to the affective characters projected onto the imagined scene, due to the atmospherization of the mood in the *Bildobjekt* (PIA 25). Here, we have the *Spaltung* between the *Phantasieleib* (and, eventually, the institution of the imagination on the basis of *phantasia*) and the part of it that is atmospherized as the correlate of the mood and is at work in a pathological empathy. Finally, Richir's interpretation of case 3 consists in developing the germ folded in Husserl's analysis of a pathology of imagination and affectivity that is also of empathy. At this point, Richir unpacks the hypothesis about the origin of the unconscious that he had already sketched through his genetic explanation of the non-figured mood. This hypothesis rests on the question concerning the relationship between the mood and the imagined scene, for which I feel the former effectively but do not know why it is awakened by the latter, or, in other words, concerning where the awakened *Stimmung* may come from, which Richir describes as the question of the (link of) significativity of the mood itself. As we know, Richir had responded to this question by arguing that the non-figured mood that transfers its character to the imagined scene may have passed into the *Bildobjekt* (as a colored filter) and entailed the atmospherization of the I intentionally implicated with it (namely, the *Phantomleib*). Therefore, the new genetic hypothesis reads:

We should ask thus if, also in case 3, there has not already been a beginning of *Spaltung* between the affect that is properly lived, whose link of significativity with the imagined scene (object) escapes, and the scene itself: certainly, one or more imagined scenes “move” the actual I *in its affective lived experience*, but this may be while it does not know *eo ipso* neither why nor how [...] the paradox is that at least a part of the significativity of the lived affectivity of the I (of this or that really lived affect) escapes the act of imagination that figures the scene (object) where this part, not figured in itself, cannot be found, as if there were a “second” life “behind” the effectively lived life (in the act of imagining), but a “second” life of whom we know that it is not precisely the life of the imagination (PIA 27).

Here, we have another kind of second life that exceeds the actual life, which is not the lived experience of the imaginary I. This second life is linked to the atmospherization of the mood and its correlate in the *Bildobjekt*. It is for this reason that, in this case, Richir speaks of the “unconscious” and not the absolute “non-positionality” of the *Bildobjekt* and refers specifically to the latter's part that is “invested by the significativities lost as such as they are not figured and yet felt in the affect” (PIA 28). From Richir's perspective, we are still at the beginning of the *Spaltung* and are not yet in the field of the psychoanalytic unconscious since traumatic affectivity is required for that. However, we are already in a situation where we are unable to explain our affect before an imagined scene and, thus, we undergo a duality of life and lived experiences.

### 2.3. Pathologies of empathy

As we saw, for Richir, case 3 is also a situation of pathological empathy because we have a *Spaltung* between the *Phantasieleib* that, within the institution of imagination, responds to the life and lived experiences of the other (even if it is a figured character, within certain limits) and the *Phantomleib* (as the correlate of the mood belonging to the *Bildobjekt*). In other words, imaginative intentionalities are at work here but no longer on the phenomenological basis of *phantasia*. They are the a-subjective imaginative intentionalities, cleared of intuitive objects, that float in the *Phantomleib*. As Richir puts it, “we have there, so to speak, in the institution of imagination, a possible autonomization or emancipation of imagination in relation to its phenomenological basis of *phantasia*” (PIA 33).^16^ It is this autonomization, which entails the *Spaltung* of the *Phantomleib*, that makes the empathy carried out in the institution of imagination (quasi-empathy), pathological. In this section, I explain that Richir unpacks this insight by taking up once again cases 1 and 3 and re-examining them, this time, in light of the phenomenology of intersubjectivity that, as we saw, he had sketched in PE [through a close reading of Hua XIII (28), texts 10 and 11] and that he develops further in the subsequent sections of PIA where he takes account of the case of the missed encounter with the other (270–85).

Prior to engaging in the analysis of the pathologies of empathy or the pathological at work in quasi-empathy, Richir offers us a recapitulation of the two key features of his phenomenology of intersubjectivity, which I summarize here. First, there is an element of narcissism, that is, of projection or duplication, in the empathy carried out in/by imagination. Second, the real encounter can take place only when mimetic *phantasia* is put to work as it responds from within and in an active and non-specular (or non-narcissistic) way to the phenomenological blinking of the *Leib* in the *Leibkörper* of the other (20). In the text under scrutiny here, Richir reproduces these features in contrast between the real encounter with the other and the missed one. In the first case, through empathy, we have a feeling for the other (even if figured, as we know). In the second case, we (pre-) suppose the other's lived experiences, through a quasi- or imaginary empathy (PIA 29–30). Richir starts by looking into the real encounter with the other:

In the effective encounter, the *phantasia* that is called into play through the *Phantasieleib* blinking in the *Leib*, and in that which the *Leibkörper* retains of the *Leib*, is what allows the primordial *Leib* (that is an absolute here) to encounter with another absolute here where it is not in reality but from where it can seize this other absolute here, from *inside*, through *phantasia*: this is precisely the beginning of that which we call a non-specular and active *mimesis* from within (since the *Leib* and the *Phantasieleib* are unfigurable) (PIA 30).

Here, Richir identifies two moments in the activity of *phantasia*. The latter is initially non-positional and non-figurative. Non-positional as I can grasp something of the lived experiences of the other not by positing myself in the place of the other but by responding to the other's *Leib*, blinking through its expressions, from my *Leib*, an activity that is designated in PE as the mimesis (not the duplication) of the *Leib* by itself (PE 285). As Richir puts it here, “I am there not with my *Leibkorper*, but, from my *Leiblichkeit* (my *Phantasieleiblichkeit*), in *phantasia*” (PIA 31). Therefore, *phantasia* is also non-figurative since the other's life is, in principle, inaccessible to my intuition *in the present*: “it is this non-figurability in an intuitable object (and fulfilling an objective intuition) that does not only exclude that it be seized in an image of imagination [...] but rather requires *phantasia*” (PIA 31). Later, for Richir, *phantasia* becomes exceptionally positional as it develops into the apperception of the other's *Leibkörper* and, consequently, into my individuation. *Phantasia* situates or anchors the *Leib* of the other in the other's *Leibkörper* and, in doing so, also makes my *Leib* appear as in turn situated and anchored in my *Leibkörper*. Here, we have the institution of effective intersubjectivity. Finally, *phantasia* is transposed into the sensation or feeling of the inside of the other's *Leib*, namely empathy, which as we know is non-figurative, or an intuition of what is non-figurable (PIA 30). From this, Richir dramatically concludes that there is no room left for the institution of imagination in the real encounter with the other. This institution can occur later, not in the presentification of the lived experience of the other, which, as we know, presupposes mimetic *phantasia*, but of “the lived experience of the encounter with the other,” that is, “of the sense or senses that are sketched or made in the encounter itself, in the double, non-specular mimesis from within, carried out from both sides, from my *phantasia* and that of the other” (PIA 31). Here, Richir introduces the distinction between the *Sinnbildung*, which accounts for the register of the sense in the making in the real encounter, and the *Sinnstiftung*, which implies the institution of the imagination on the basis of this encounter. It is worth recalling that, for Richir, the real encounter with the other through the mimetic *phantasia* constitutes the genetic condition for the transmission of sense among humans. As Richir puts it here, “sense in the making... is *eo ipso* intersubjective” (PIA 31). Now, how is imagination instituted in this situation of effective intersubjectivity? Again, I recall that this institution consists in the fugitive arrest, due to a not necessarily traumatic excess of affectivity, in an intentional present, of the discontinuous temporalization (in the presence without an assignable present) that is distinctive of the apparitions of *phantasia*, and that the real encounter in which sense makes itself amounts to these apparitions (PE 72–143). Therefore, Richir suggests that the *Sinnstiftung* takes place when *something* of what is going on between us, in the encounter, flows “in the intentional present of the act of imagination, in order to be deposited in habits and sedimented senses, before seizing it again in remembering” (PIA 31).

The imagination instituted here does not seem to be autonomized from its basis of *phantasia*, nor to be atmospherized in the *Phantomleib*. It does not seem to be narcissistic or pathological, in principle. For a pathological *Einfühlung* to develop, specific imaginative intentionalities must be at play. Richir identifies this pathological situation with that of having an image of the other, whether it is by imagination or fixed on a physical support: “when the other is figured in imagination without being there *leiblich*, that is, without being in presence in the *Leiblichkeit* of its apperceptible *Leibkörper*” (PIA 31). In this case, Richir explains that there is no empathy, no transposition of *phantasia* into the feeling of the lived experience of the other. We may wonder whether this means that there is no empathy at all through figuration. However, as Richir has already remarked and further explains later (497–508), in the wake of Husserl, there can be empathy also with figured characters when the figuration implies the mimetic activity of *phantasia* and calls the latter into play in the spectator. In other words, in this case, even if in a figured scene, there is still a *leiblich* presence of the other. Richir's argument is that, when I have an image of the other, the *Spaltung* has already begun: I have a mood that belongs in the *Bildobjekt* (case 3) and, most likely, I dive into the latter (case 1) and end up grasping, of the other, only that which I suppose about its lived experience, namely I daydream. Richir builds on this argument to advance his last and most general hypothesis about the genesis of the unconscious, which places the latter in the articulation of daydreaming and traumatic affectivity. To unfold his argument, Richir focuses on the two features of the image of the other that I have, *Bildobjekt* and *Bildsujet*. First, there is no temporalization of the other (here *Bildsujet*), in presence, that can be responded through another temporalization in presence, namely the mimetic activity of *phantasia*, and thus, we do not find ourselves in the effectively intersubjective situation of sense-making (*Sinnbildung*). Conversely, the lived experience of the encounter (*Sinnstiftung*), and not the life of the other, has already been caught in the intentional present of an act of imagination, which can only be taken up again in another intentional present by another act of imagination. As Richir points out:

Since then the “living” imagination or portrait are as such because this fixation opens behind them onto a *supposed* past and before them onto a future that is equally presumed (there is often the aliment of a *told* legend or story, because of this supposition or presumption), which, however, lost as they are with regard to the presence without the present of their sense in the making, and distinguished from the retentions and protentions of the intentional present of imagination, can only be taken up through other fixations of the imagination, through other intentional presents (PIA 32).

Furthermore, Richir remarks that the figured gesture in which the other's life is arrested has “something theatrical, which proceeds from representation or staging” (PIA 32). Here, I highlight that the theatrical element should be understood as preventing the mimetic *phantasia* from going into action and, more likely, as lending itself to the already made stories that I tell myself while facing the image of the other. It is at this point precisely that Richir interweaves the situation under scrutiny with the cases of the *Spaltung* examined above. This is also the point where we move from the examination of the phenomenological status of the *Bildsujet*, in the case that I have an image of the other, to that of the *Bildobjekt*, which, as we know, is the site of the pathologies of the imagination and affectivity. We start from case 3, given that I have this or that feeling in front of the figured life of the other. As Richir explains, “I ‘feel' there the present of the lived experience of the other presentified by imagination only by ‘instants'... like in case 3” (PIA 32), but we can also move to case 1, by diving into daydreaming. Thus, in Richir's words, “I remind myself of the history that is told or that I imagine and, consequently, I imagine the lived experiences and the life of the I-other or the other... figured like a *Bildsujet*” (PIA 32). Here, Richir seems to suggest that the quasi-empathy is pathological to the extent that, since there is no index of the other's life in the latter's figuration in images, that is, in the other as a *Bildsujet*, my feeling of the other proceeds from the *Bildobjekt* and has as its correlate a-subjective imaginative intentionalities that are cut from the quasi-position of imagination. We know that this feeling initially consists of (i) an affectivity, more precisely, a mood, that is effectively felt before the image and operates like a colored filter, and, eventually, later, (ii) an imagined affectivity that occurs in my daydreaming.

Therefore, we find again, in the pathological empathy, the global structure of the *Spaltung* understood as the I's passing or diving into the *Bildobjekt* and characterized by the circulation of affectivity between cases 3 and 1. Richir describes this structure as a two-step movement. First, he remarks, “I imagine by starting from the affective significativities depending on the *Bildobjekt*” (PIA 32). Later, he goes on, “through that, I can pass into the scene by imagining what the other imagine and would live” (PIA 33). As we know from Husserl, the imaginary life carried out in the *Bildobjekt* can be accomplished in and by consciousness as a fiction. But can it also remain unaccomplished: on which condition? Richir addresses these questions in an implicit way by formulating his general hypothesis about the unconscious precisely in relation to the global structure of the *Spaltung*. What makes the latter the origin of the unconscious and thus in the properly called *Spaltung* of consciousness is the transition of the imaginary life in the *Bildobjekt* into the psychoanalytic unconscious explored by Freud. Here, Richir explicitly affirms that, as I have anticipated, this transition is due to an excess of affectivity (in a psychoanalytic lexicon, to traumatic affectivity):

This life, thus, cannot escape the awake consciousness, this is the proper case of the *Spaltung* of consciousness, when these fictive lived experiences, as Husserl would put it, are properly unaccomplished and, moreover, we will see them coupled with one or more excesses of affectivity: they remain buried in the *Phantomleib* to which a *Phantom-Ich* corresponds, this is what Freud first calls, with Breuer, “hypnotic states,” and later, by himself, “unconscious thoughts.” (PIA 33).

It is worth remarking that here, Richir subscribes to Donald Winnicott's radicalization of Freud's conception of affective traumatism, which he explores more in-depth later. By tracing the origin of the *Spaltung* and the unconscious back to affective traumatism, on the one hand, Richir acknowledges Freud's discovery that it is only through affectivity that the *Spaltung* can be phenomenologically attested (PIA 39). On the other hand, Richir suggests that this traumatism is at work since the first encounter with the other, that of the infant's relationship with its mother, as Winnicott demonstrates in his studies on the archaic anxiety that may develop from this relation [326–29; (30)]. Now, the genetic hypothesis formalized in the passage under scrutiny does not only bring Husserl's phenomenology of imagination further by articulating its implications with the study of psychoanalysis. It also sketches a non-standard phenomenological interpretation of the Freudian unconscious, which is fully developed in PIA Section III (chapter 1). As Richir observes, “what counters the return [of daydreaming] to accomplishment... is not repression” (PIA 33). This interpretation, which I cannot discuss here, confirms that, for Richir, as we have seen so far, there is no subject in action in the genetic process of the *Spaltung*, neither in the case of the pathologies of imagination and empathy nor in that of the transition to unconscious thoughts.^17^

## 3. Conclusion

In this article, I have focused on the genetic analyses through which Richir, by building on Hua XXIII text n.16, searches for the beginning of the *Spaltung*, namely the pathological element in the articulation of imagination and affectivity that undergirds the origin of the psychoanalytic unconscious. In doing so, I have attempted to highlight the contribution that these analyses make to the research on imagination disorders that authors such as Rasmussen and Parnas have carried out recently. In what follows, I would like to summarize the main features of this contribution, which, as I anticipated, hinges on a complexification of the standard phenomenological model of consciousness employed by those authors. First, by subscribing to Richir's distinction between *phantasia* and *Sinnbildung*, on the one hand, and imagination and *Sinnstiftung*, on the other hand, we discover that the pathological of the imagination consists in the latter's autonomization from the phenomenological basis of *phantasia*, whose privileged site is affectivity since that autonomization takes place when a mood atmospherized in the *Bildobjekt* transfers its character to the imagined object without an evident link of significativity. In this case, as we know, we have the corresponding atmospherization of the I, the springing of a-subjective imaginative intentionalities, and so forth. Second, the pathologies of imagination are also of empathy, precisely because the atmospherized mood operates like a filter in the image that I have of the other. Third, affectivity plays a key role at the beginning of these pathologies: as the atmospherized mood that I have evoked above and as the traumatic affectivity which makes the transition to the unconscious and the fixation of the phantasm possible. Ultimately, Richir's genetic analyses build a phenomenological framework for the critical rereading of the psychoanalytic account of the fixation of the phantasm, which Richir unfolds later and of which I have limited myself to giving a few indices here.

## Author contributions

The author confirms being the sole contributor of this work and has approved it for publication.

## Footnotes

^1^All translations from Richir's works in this article are mine.

^2^Identified as one of the three key figures of *Neue Phänomenologie* in France, with Michel Henry and Jean-Luc Marion [see Gondek and Tengelyi (9)], as Alexander Schnell argues, perhaps more justly, Richir stands as a singular figure in the phenomenological tradition *tout court*, for his original engagement with this tradition (2011). For an introduction to this figure, see Richir and Carlson (10).

^3^For concepts such institution and transposition, that are characteristic of Richir's project of a non-standard recasting/refoundation of Husserl's genetic phenomenology, see the final methodological section of *Phénoménologie en esquisses* (PE 457–506). More specifically, on the architectonic transposition of *phantasia* in imagination, which Husserl designates as *phenomenization* (PICM 77–80), see PE 77–84, and the following related scholarship: Schnell (13); Carlson (14), and Posada Varela (15).

^4^These pathologies are designated as “transcendental” (PIA 34) since they are examined, from the perspective of their conditions of possibility, as moments in the transcendental history of the concrete I. As they deal with these moments, the analyses carried out in PIA constitute an extension of the genetic account of the so-called normal life of consciousness carried out in PE.

^5^For an in-depth interpretation of this articulation, see Fazakas (16). This text also offers a summary of Richir's reading of Hua XXIII text n.16, which I examine below in a more extended and focused way. Overall, on the articulation of phenomenology and psychoanalysis in Richir's work, see Mesnil (17).

^6^Richir's analyses of the pathologies of the imaginary demarcate themselves from Sartre's since they build on Husserl's text in an explicit and direct way. On Sartre's analyses, see (19), 121–50.

^7^Richir explains that he employs “a little abusively” the term “phantasm” to designate the structure of what Freud calls *Phantasie* (which is not Husserl's *Phantasie*): “a scene imagined according to a scenario that is more or less fixed” in which the imagining I can find itself in one of the two cases under scrutiny here (neuroses or perversions; PIA 39).

^8^Richir goes back to text n.16 and the related topics in a later short essay, which aims to be a comprehensive reading of Husserl's phenomenology of *phantasia* [see Richir (20)].

^9^On the phenomenology of affectivity, see Fuchs (21). On the problem of fictional emotions, see Cavallaro (22).

^10^For a “standard” (from Richir's perspective) reading of the relations among image consciousness, *phantasia*, and imagination, see Bernet (23), 75–117, and de Warren (24), 143–67.

^11^As Richir puts it, “the question that arises here is whether this absence of position makes the fictive I *blind* to the imagined [and thus its life excessive with respect to that of the actual imagining I] or it signifies a quasi-position of the imagined” (PIA 13).

^12^On this, see (26), 44, where the fascination of my thoughts is seen as a feature of captive consciousness (that is, the consciousness pathologically captured in its imagining attitude); and Blanchot (27), 27–8, which describes fascination as the passion for the image.

^13^Here, we may wonder whether there is a certain affinity between these intentionalities and the captive consciousness mentioned in the note above.

^14^Richir's treatment of the primary process, whose work he identifies as the 1st degree passive syntheses (as distinguished from the more archaic and properly phenomenological 2nd and 3rd degrees passive syntheses), deserves a detailed examination, which cannot be developed here. For this treatment, in relation to Freud's *Traumdeutung* and in a psychopathological context, see PE 311–21 and PIA 335–46, respectively.

^15^On Husserl's analysis of the coloration associated with the mood, see Zirión Quijano (29).

^16^For a more focused description of the *Stiftung* of this special a-subjective imagination, see PIA 433–34.

^17^Later, by commenting on a 1908 text by Freud (“On hysterical phantasms and their relation to bisexuality”), Richir argues that, alternatively to what Freud seems to suggest therein, the *Spaltung*, and not repression, is the cause of the fall into the unconscious. Ultimately, the latter is understood as being independent from the active or voluntary part of the subject (PIA 296–7).
